# A Spatial Survival Model for Risk Factors of Under-Five Child Mortality in Kenya

**DOI:** 10.3390/ijerph19010399

**Published:** 2021-12-30

**Authors:** Kilemi Daniel, Nelson Owuor Onyango, Rachel Jelagat Sarguta

**Affiliations:** School of Mathematics, University of Nairobi, Nairobi 30197, Kenya; onyango@uonbi.ac.ke (N.O.O.); rsarguta@uonbi.ac.ke (R.J.S.)

**Keywords:** under five child mortality, kenya dhs, spatial survival models, bayesian survival applications

## Abstract

Child mortality is high in Sub-Saharan Africa compared to other regions in the world. In Kenya, the risk of mortality is assumed to vary from county to county due to diversity in socio-economic and even climatic factors. Recently, the country was split into 47 different administrative regions called counties, and health care was delegated to those county governments, further aggravating the spatial differences in health care from county to county. The goal of this study is to evaluate the effects of spatial variation in under-five mortality in Kenya. Data from the Kenya Demographic Health Survey (KDHS-2014) consisting the newly introduced counties was used to analyze this risk. Using a spatial Cox Proportional Hazard model, an Intrinsic Conditional Autoregressive Model (ICAR) was fitted to account for the spatial variation among the counties in the country while the Cox model was used to model the risk factors associated with the time to death of a child. Inference regarding the risk factors and the spatial variation was made in a Bayesian setup based on the Markov Chain Monte Carlo (MCMC) technique to provide posterior estimates. The paper indicate the spatial disparities that exist in the country regarding child mortality in Kenya. The specific counties have mortality rates that are county-specific, although neighboring counties have similar hazards for death of a child. Counties in the central Kenya region were shown to have the highest hazard of death, while those from the western region had the lowest hazard of death. Demographic factors such as the sex of the child and sex of the household head, as well as social economic factors, such as the level of education, accounted for the most variation when spatial differences were factored in. The spatial Cox proportional hazard frailty model performed better compared to the non-spatial non-frailty model. These findings can help the country to plan health care interventions at a subnational level and guide social and health policies by ensuring that counties with a higher risk of Under Five Child Mortality (U5CM) are considered differently from counties experiencing a lower risk of death.

## 1. Introduction

The burden of mortality in children has remained a key area of concern for nations and organizations in the world. The year 2018 recorded approximately 5.3 million children and infant deaths worldwide. The risk of under-five mortality in the WHO Africa region was 76 deaths per 1000 live births, which was eigth-times higher than the WHO European region [1]. This is far from ideal and is a worrying situation. The rate of mortality in Kenya in the same period was 46.7 deaths per 1000 live births. This was above the Millennium Development Goal target expected by 2015 of 33 deaths per 1000 live births, and the 2030 goal of 25 deaths per 1000 live births [2].

This national estimate, worrying as it is, is also accompanied by different concealed spatial variations [3] and, therefore, poses a complex problem unravelling the intricate localized or sub-regional variations in an attempt to better understand and offer feasible solutions to this burden. There exist various causes of child mortality in Kenya. The framework outlined by [4] provides a blue-print on studying mortality in third world countries.

Key sources of variation in child mortality in Africa include individual characteristics, such as wealth index, level of education, and nutrition [5]. The duration of breastfeeding, use of antenatal care, maternal health care services, birth intervals, and the age of the mother have also been known to be significant in determining the mortality of a child [6,7,8,9]. In addition, maternal education, a social economic factor, the presence of it and lack thereof has been shown to significantly affect the rate of survival of a child [10]. Other areas of concern on the determinants of child mortality include cultural practices [11].

The existence of these variations caused by the risk factors above are known to vary from region to region in a country. This suggests a presence of community level characteristics that influence health outcomes [12]. Such community level variables include income, place of residence, infrastructure, and region among others. These variables influence behaviors and patterns regarding child and maternal health care, and are significant in explaining child mortality [13]. Such disparity could be at the country level or county level. Urban areas have had lower risk of death for infants and children [14] in Ethiopia and yet higher odds of death in Nairobi [2] compared to rural areas.

It is, therefore, important to understand the distribution of these factors geographically and their influence in various locations. In order to understand these disparities, and eventually the risk factors, survival methods have been applied as a critical tool in the analysis of life data [14]. Cox models have been applied to model the time to occurrence of an event [15] and showed significant improvements compared to classical regression techniques [3].

The assumption of Cox models is that child survival is dependent on a baseline survival and certain risk factors; however, this is not often true in reality as survival data are dependent when clusters or locations are considered. This dependency introduces unobserved random effects (frailties) present at various levels, and suggests the presence of community level characteristics that influence health outcomes [12]. The existence of these effects caused either by a location, or a presence in certain population clusters leads to the use of spatial survival models to capture these unobserved effects, especially if they are geographical.

In spatial survival analysis, the survival of a child is assumed to be correlated if areas or points are close together, and it is possible to analyze data that are clustered when the clustering is a result of geographical regions or some other form of stratification [16]. The expected hazard rates will be more similar in neighboring regions, owing to underlying factors, such as access to health care services, which varies spatially. Studies, such as Kazembe et al. (2012), Ezra et al. (2016), Hesam et al. (2018), and Kazembe et al. (2007), in various regions in Africa emphasized the significance or improvement that spatial survival models have over and above the existing survival analysis models, particularly when the spatial heterogeneity is significant [3,17,18,19].

The purpose of this study is to evaluate the effect of spatial variation on under-five mortality in Kenya. This is informed by the introduction of counties in 2010 and the delegation of health care functions to the counties. The extent of the disparities in the burden of child mortality between those counties is not known, and therefore it is important to investigate the differences to aid in county-based health care planning and intervention. This is done using a spatial Cox Proportional Hazards model using the Kenya Demographic and Health Survey, (2014) data set.

## 2. Materials and Methods

### 2.1. Data Source

The data analyzed in this study were sourced from the Kenya Demographic and Health Survey 2014 [20]. Authorization to use the data set was obtained through the DHS Portal, which contains a repository of the survey data. The Demographic and Health Survey is an initiative sponsored by the United States Agency for International Development (USAID) in partnership with other Kenyan research agencies, including the Kenya National Bureau of Statistics (KNBS), National Council for Population and Development (NCPD), Ministry of Health, Kenya Medical Research Institute (KEMRI), and National AIDS Control Council (NACC). The DHS program has been run in many nations to provide periodic updates, outlooks, and estimates of various indicators, such as maternal and child health and individual level information pertaining to the health of such individuals in specific cases. The data collected contains information about families, children, child bearing mothers, and other socioeconomic factors as relevant to help monitor the population and health status in Kenya.

### 2.2. Survey Design

The 2014 KDHS survey was the first of its kind to provide county level estimates since the promulgation of the New Constitution in 2010. County information was included in the dataset using a separate country shape file. The Kenyan agencies provided the personnel to conduct the survey. The survey used samples from the country’s population and housing census estimates. Using a two stage sampling frame, Enumeration Areas (EAs) in the census served as the primary sampling unit. Clusters were selected in the first stage from the Enumeration Areas, while households were selected in the second stage. All mothers aged 15–49 years old were eligible respondents, and information about children born 5 years prior to the survey was obtained.

### 2.3. Variable Selection

The variables used in the study were pre-selected based on the existing literature on the significant determinants of child mortality. The variables ranged from demographic and socieconomic factors to variables specific to the mother and child. The study focused on the existence of spatial variations and the differences in child mortality across regions in the country. The demographic and socioeconomic variables selected included the sex of the child at birth, maternal age at birth, age of respondent at first birth, gender of the household head, Wealth Index, Highest Education level, and the type of place of residence. The geo-referenced regions were the counties and coordinates (displaced) provided. The primary outcome was the mortality of a child, defined as the time to death of a baby before his or her fifth birthday.

### 2.4. Statistical Analysis

Statistical analyses were performed using the R-software for statistical computing. Spatial survival analysis to estimate the spatial differences in mortality across the counties was performed using Intrinsic Conditional Autoregressive Models incorporated into the Cox proportional hazard model. In addition, the effects of the preselected variables were estimated using a Cox proportional hazards model.

We compared two survival analysis models, one with a spatial frailty assumption, while the other one had no frailty assumption. Model comparison was done through the Deviance Information Criterion. For an estimation of the model parameters, a Bayesian approach was used and assigned priors for the model priors and the distribution of the baseline survival function. An Intrinsic Conditional auto-regressive (ICAR) prior was assumed to model the spatial structure [3]. The results are presented as posterior estimates of the spatial variance and the covariates and the corresponding 95% Credible Intervals. For more details on the methods please refer to Appendix A.

## 3. Results

**Figure 1:**
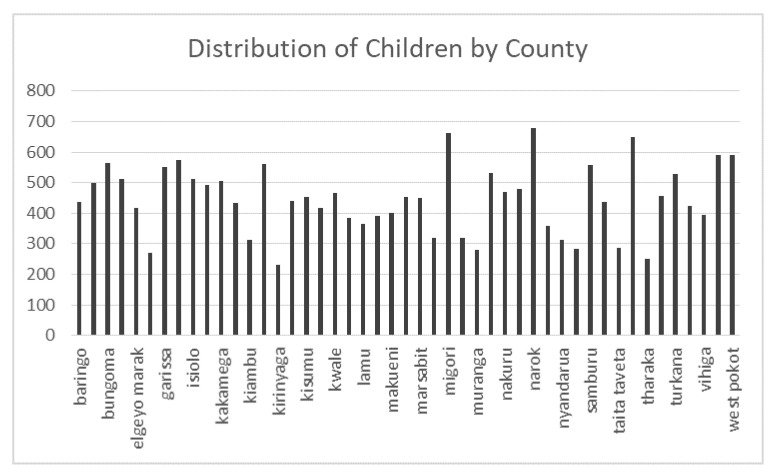
Distribution of children by county.

The number of children in each county is shown in Figure 1. The sample was representative of the children in each county, due to the sampling frame.

### 3.1. Model Comparison

This section deals with the comparison of the two models fitted, the spatial differences in the risk of child mortality, and the results of the covariates considered. The Deviance Information Criterion was used to compare the performance of the two models, where a lower DIC indicated a better model fit on 856 degrees of freedom. The first model was fitted using the Proportional Hazards assumption on the child mortality data, where the response was the hazard of death dependent only on the baseline hazard and a set of covariates. The DIC value for this model (3384) was larger compared to the spatial model as shown in Table 1.

The second model was fitted with the spatial frailty term and had the lowest value of the Deviance Information Criterion (3344) and, therefore, a better fit. This model assumed unobserved variation (heterogeneity) at the geographical (county) regions in the country. There was a significant improvement brought about by the spatial model with frailties that accounted for randomness apart from the independent covariates. The Watanabe Akaike Information Criterion deals with the predictive power of the model, where lower values mean higher predictive power. The Spatial Cox proportional hazards model had the higher predictive power as shown in Table 1.

### 3.2. Goodness of Fit Tests

The Markov Chain Monte Carlo (MCMC) estimation deals with estimating model parameters in a Bayesian setting. The MCMC sampler explores the parameter space of a certain parameter, resulting from prior knowledge about the parameter and the likelihood of the observed values. To ensure mixing, the resulting chain selection of parameters results in a stationary distribution of the parameters. Trace plots—time plots of the Markov Chain—were used to assess the proper mixing of the parameters and were stationary, indicating convergence, and hence were reliable.

**Figure 2:**
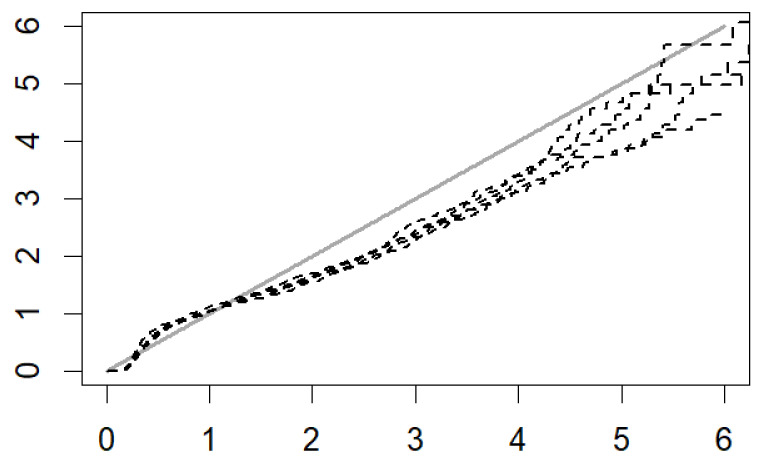
Cox–Snell plot.

To assess the goodness of fit of the model, a Cox–Snell plot was used. Cox–Snell residuals; the residuals of the observed data points and the predicted values analogous to normal probability plots test the reliability of the model. The Cox–Snell plot showed hazard plots that were approximately straight with slope one, indicating a good model fit, as shown in Figure 2.

### 3.3. Model Results

The spatial frailty model had the best fit on the data. The posterior variance of the ICAR frailty; accounting for the variance due to the spatial effect is shown in Table 2. The mean was 0.2001, which was statistically significant with a 95% credibility interval (0.0578, 0.4649). On average, the spatial effect explained 20% of the heterogeneity variance.

The statistically significant determinants of child mortality included the sex of the child, age of the respondent at first birth, gender of the household head, and whether a family had multiple sets of twins. These encompass the set of demographic risk factors associated with child survival, while the socio-economic factors associated with child survival included the highest level of education (secondary). The posterior mean for the sex of the child was −0.1304, and the median was −0.1315. The hazard ratio was exp(−0.1304) = 0.877, and the probability of survival was higher in female children by 12.23 percent compared to male children, holding all other predictors constant. The 95 percent credible interval of the hazard ratio was (0.7761, 0.9921).

The mean for the sex of the household head was −0.1550 and the median was −0.1579. Adjusting for the sex of the child, level of education, and age of the respondent at first birth; for a child born in female-headed households, the computed hazard was 0.8564, and therefore the probability of survival increased by 14.36 percent compared to male-headed households. The age of a respondent at first birth had a mean and median of 0.025. There was a positive association, i.e., an increased risk of death for children for an increase in age at first birth. There was also an increase of risk of death by 2.5 percent for a unit increase in age of a respondent at first birth. Families with multiple sets of twins (the first multiple of twins refers to the first set of twins born, while second multiple of twins refers to a second set of twins) had a high risk of child mortality. The mean was 0.4438, with a hazard ratio of 1.5586. Children born as a second set of twins had a higher risk of death by 55.86 percent compared to children born in single births.

The respondent’s level of education was statistically significant at the secondary level. Respondents who had attained a secondary level of education in Kenya had a mean of −0.2982 and a hazard ratio of 0.7422; the risk of child mortality in respondents who had attained secondary education decreased by 25 percent compared to respondents who had not had any education at all. These results are shown in Table 3.

Table 2 shows the posterior frailties on the variance of the spatial term. The variance was 0.2001 and was significant at 0.05, the credible interval was (0.05776, 0.4649). Adjusting for the effect of the covariates, there were other unobserved spatially varying covariates relevant for child survival in Kenya.

**Figure 3:**
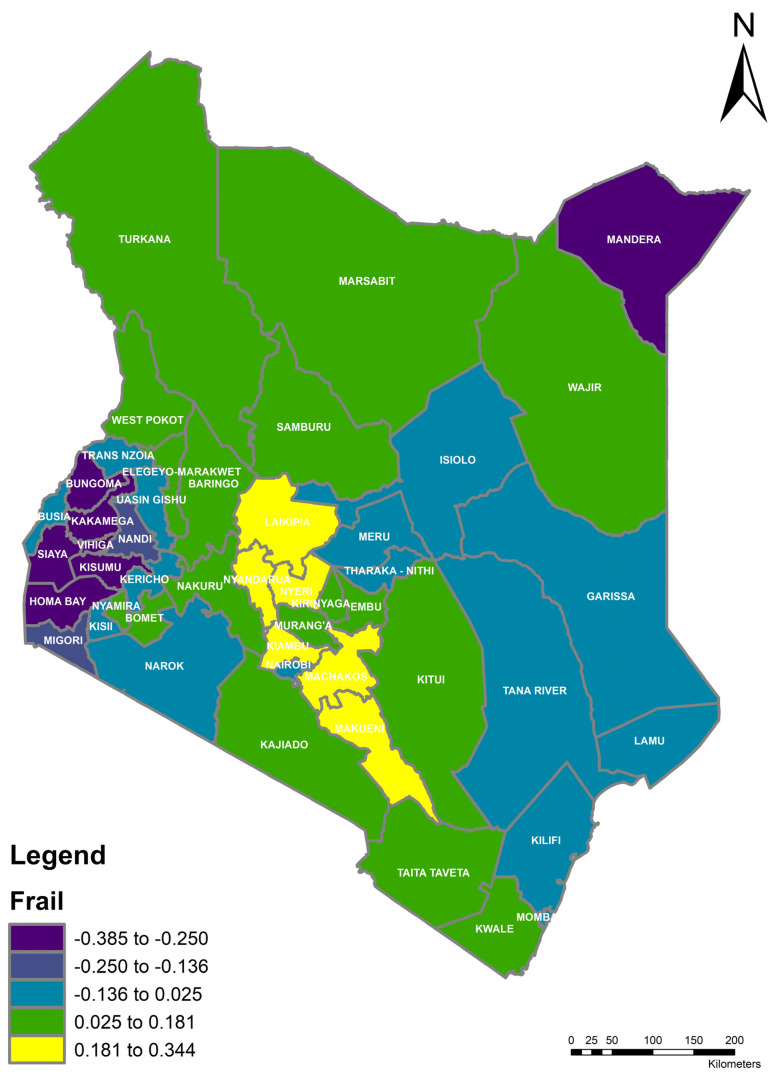
Map of posterior frailties. Kenya is a country in Africa on the East African region at (4.0889, 39.7707) divided in half by the Equator.

In Figure 3, we can identify a cluster of counties with higher median frailties or a higher hazard (counties with yellow colors) located centrally in the country. This shows that these counties, apart from the covariates, have a spatial correlation, hence, the similarities in the hazard of death for a child. Children from these counties (Makueni, Machakos, Kiambu, Nyandarua, Nyeri, and Laikipia) were shown to have the highest risk of mortality adjusted for effects of covariates. Medium level frailties were identified as clustered around the Garissa, Tana River, Kilifi, and Lamu counties, Meru and Tharaka-Nithi counties, counties in Rift Valley (Nandi, Uasin Gishu, and Elgeyo Marakwet), and Narok and Kisii counties.

Counties in Western Kenya and Nyanza also had low frailties with spatial correlation (counties with purple color) related to death from other causes apart from the main effects of the independent variables. The highest number of counties, however, (counties with green color) had the same frailties and a similar hazard. These were counties in Northern Kenya, South Rift, and counties bordering Tanzania on the South West. The others were around the Mount Kenya region (Kirinyaga, Embu, and Murang’a) that showed a homogeneous risk of death of a child.

## 4. Discussion

The aim of the study was to bring out the spatial disparities on child mortality existing in the counties in Kenya. The spatial frailty proportional hazards model was used to determine the risk factors associated with under-five mortality in the country. Two models, the proportional hazard model without any frailty assumption and a frailty model assuming variations across space were compared.

The study reveals unobserved spatially varying covariates relevant for risk factors of child mortality in addition to the risk factors explaining child mortality. The ICAR spatial survival model was used to model these unobserved effects and to model the correlation and or clustering between these regions. This study, therefore, analyzed the time to death of a child using spatial survival models. The spatial model performed better compared to the non-spatial model, similar to [19].

Using the Akaike Information Criterion (AIC), the Cox Proportional Hazards model with spatial frailties performed better than the non-frailty model. The predictive ability of the spatial model was also higher compared to the non-spatial non-frailty model. The variation introduced spatially significantly improved the model, making it the superior model. The better fit model was therefore used together with the covariates to investigate the underlying factors contributing to child mortality.

The results show that patterns of child mortality in Kenya have a spatial structure, which is similar for the neighboring counties around Central Kenya, although majority of the counties appear to have similar frailties and, similar spatial distribution. The presence of unexplained variation, save for the main effects, was statistically significant and improved the model. This may be explained by similarities in access to healthcare and other amenities and the variance in counties, similar to [3].

After adjusting for the spatial structure, gender of the child, and respondent’s age at birth, the probability of survival of a child decreased with an increase in the age at first birth. Multiple births were found to be significant risk factors associated with child mortality. The gender of the child (male children had a high risk of death) and whether a child was in a multiple birth (twins) were important risk factors (also found by [3]) associated with child mortality. The gender of the house-hold head was also found to be significant. The level of education, also found by [8], was an important risk factor for child mortality.

There is, therefore, an increased survival probability for children born to families whose respondents have attained a secondary level education. Children from mothers who had attained secondary education had higher chances of survival compared to children from mothers who had no education or only primary education. A primary level of education as well as a higher education level (beyond secondary) were not significant risk factors associated with child mortality. It is important, therefore, for most mothers to attain a secondary education level in the country for a reduction in the risk of child mortality.

## 5. Conclusions

This study brings out the spatial disparities that exist in the country on child mortality in Kenya. From the models used, the model incorporating spatial terms performed better; hence, there is a need to consider using the improved model to model key outcomes, such as the gender of the child, the age of the respondent at first birth, whether a child is in a multiple birth, gender of the household head and level of education, and other country indicators of health in the future.

The specific counties have mortality rates that are county-specific, although neighboring counties have similar hazards of death of a child indicating relationships of a county and its neighbour. It is important, therefore, to consider interventions that take into consideration the effect of where a child is born from (county) when providing intervention to reduce the risk of mortality.

In addition, such interventions should pay attention to the case where a child is born in a multiple birth and should provide special care and monitoring in families where such cases occur. Families with histories of multiple births, either twins or triplets and so on should have a flag on them, such as governmental, county, or community level initiatives that offer extra care for such children.

The important of education cannot be over-emphasized to ensure a child is kept safe from mortality. This ensures mothers have appropriate information on diets with more nutritional value, breastfeeding, immunization, and hygiene among others. Mothers with some level of education (secondary) are also more likely to have a form of income compared to mothers with no level of education. A level of education for a mother also implies that one is more likely to be married by a husband with similar or higher levels of education, which increases income for the overall household and, hence, significantly improves the probability of survival for a child. It is important, therefore, for most mothers to attain secondary education level in the country for a reduction in the risk of child mortality.

As the country aims to achieve the stipulations of the Millenium Development Goals, as it failed to hit the set targets for MDG 2015, and, in order to hit the targets for MDG 2030, the significant differences witnessed in counties should be considered at the policy level. All attempts to smoothly hit the 2030 target within the timelines should include a devolved approach, where counties spearhead the reduction in risk of mortality. Regional blocks for which mortality risks appear similar should be considered as an entity to ensure that the menace that is child mortality is dealt with.

## Figures and Tables

**Table 1:** The model comparison results.

Model	Deviance Information Criterion (DIC)	Log Pseudo Marginal Likelihood (LPML)	Watanabe-Akaike Information Criterion (WAIC)
Cox Proportional Hazards (No Frailty)	3384	−1694	3389
Proportional Hazard Spatial Frailty Model	3344	−1682	3362

**Table 2:** Variance of the ICAR frailty term.

ICAR Frailty	Mean	Median	Std Deviation	95% Lower CI	95% Upper CI
Variance	0.2001	0.1776	0.1117	0.0578	0.4649

**Table 3:** Model summary regression output.

*Variable*	Mean	Std Dev	95% CI−Low	95% CI−Upper
*Sex of the child (Female)*	−0.1304	0.0636	−0.2535	−0.0079
*Type of place of residence (Rural)*	0.1424	0.083	−0.02	0.3102
*Sex of household head (Female)*	−0.155	0.0693	−0.2813	−0.0159
*Wealth Index (Poorer)*	0.0851	0.0952	−0.0979	0.2751
*Wealth Index (Middle)*	0.1952	0.102	−0.0038	0.3824
*Wealth Index (Richer)*	0.2017	0.1188	−0.0291	0.4229
*Wealth Index (Richest)*	0.2512	0.1533	−0.0292	0.5688
*Highest Level of Education (Primary)*	−0.1756	0.1095	−0.3869	0.0499
*Highest Level of Education (Secondary)*	−0.2982	0.1385	−0.5533	−0.0128
*Highest Level of Education (Higher)*	−0.046	0.2001	−0.4231	0.3574
*Age of respondent at first birth*	0.0251	0.0112	0.0041	0.0463
*Maternal age at birth*	0.0013	0.0056	−0.0089	0.0126
*Child is twin (1st Multiple)*	0.1642	0.1421	−0.1285	0.4325
*Child is twin (2nd Multiple)*	0.4438	0.1749	0.0951	0.7585

**Figure A1:**
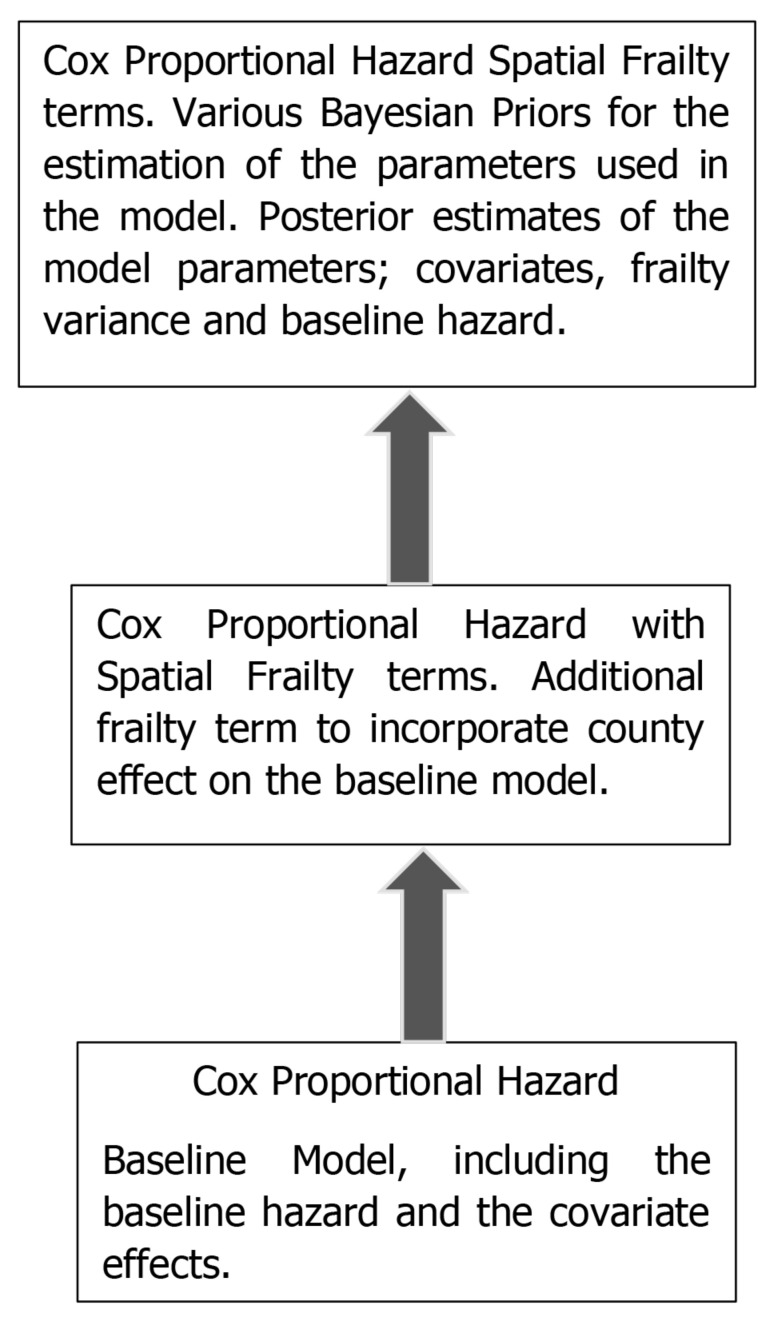
The model implementation chart.

## Data Availability

Data Available through the DHS Online Portal https://dhsprogram.com/data/, accessed on 22 June 2020.

## Abbreviations

The following abbreviations are used in this manuscript:

KDHS	Kenya Demographic and Health Survey
MCMC	Markov Chain Monte Carlo
ICAR	Intrinsic Conditional Autoregressive
U5CM	Under Five Child Mortality
WHO	World Health Organization
USAID	United States Agency for International Development
KNBS	Kenya National Bureau of Statistics
NACC	National Aids Control Council
NCPD	National Council for Population and Development
KEMRI	Kenya Medical Research Institute
MDG	Millenium Development Goals

## Appendix A. Methods

### Appendix A.1. Introduction

The event of interest was in modelling survival times: the time taken until an event of death occurred for an individual child. Assuming a sample of size *N* children aged between 0 to 59 months, the survival information of a child is $(t_{i},\delta_{i}),i\in 1,\cdots ,N$ where $t_{i}\in 1,\cdots ,60$ is the child’s survival time in months and a status variable $\delta_{i}=1$ if the child is dead and $\delta_{i}=0$ if the child is still alive [3]. Further suppose that the child is a part of a sample from *P* counties, the number of children in county *j* is $n_{i}$ where $j=1,\cdots ,n_{i}$ and $\sum_{j=1}^{P}\sum_{i=1}^{n_{i}}=N$.

### Appendix A.2. Cox Proportional Hazards Model

The proportional hazards model from [15] has a key assumption that the hazard of death for an individual is proportional to the hazard for any other individual. The hazard model dependent on the baseline hazard and a set of covariates is shown as

(A1) $$\begin{array}{c} h\left(t_{ij};x_{ij}\right)=h_{0}\left(t_{ij}\right)exp\left(\beta^{T}x_{ij}\right) \end{array}$$

where *t* is the survival time, h(t) is the hazard function, $\beta^{T}$ is a parameters vector of px1 dimension, and $x_{ij}$ is a vector with the values of the covariates in individual *i* from county j(1xp) dimension, $h_{0}$ is the baseline hazard function; while $e^{\beta_{i}}$ denotes the hazard ratios (H.R).

### Appendix A.3. Spatial Frailty Models

Let $t_{ij}$ denote the time to death or censoring for subject *i* in stratum *j* (hospital, region) $j=1,\cdots ,n_{i}$ and i=1,⋯,n, while $x_{ij}$ denotes the individual specific covariates which includes; sex of the child at birth, maternal age at birth, age of respondent at first birth, sex of household head, wealth index [20], highest education level, and the type of place of residence.

Introducing a frailty term extends the model to [21];

(A2) $$\begin{array}{c} h\left(t_{ij};x_{ij}\right)=h_{0}\left(t_{ij}\right)w_{j}e^{\sum_{i=1}^{n}\beta_{i}X_{ij}} \end{array}$$

where $w_{j}$ captures the random effect which is a random variable introduced multiplicatively to the baseline hazard. Replace $w_{j}$ with $logw_{j}$ then the frailty term is captured effectively in the exponent as shown below;

(A3) $$h\left(t_{ij};x_{ij}\right)=h_{0}\left(t_{ij}\right)e^{\sum_{i=1}^{n}\beta_{i}X_{ij}+W_{j}}$$

The term $W_{j}$ captures the differences between the stratum (clusters) that are not captured by the main effect. An example of $W_{j}$ (non-spatial) are normal random variables with mean 0 and variance $\sigma^{2}$ [21].

### Appendix A.4. Intrinsic Conditional Autoregressive (ICAR) Models

To model spatial frailties, the ICAR model was considered. The normal random variable $\phi_{i}$ accounting for the spatial interaction between regions, is conditional on the average of its neighbors; each with $\phi_{ij}$, with mean equal to the average of its neighbors $d_{i}$. The variance reduces due to an increment of the neighbors of a region, assuming a strong correlation around many neighbors. The distribution of each $\phi_{i}$ conditional on $\phi_{ij}$ and is given as;

(A4) $$\begin{array}{c} \phi_{i}|\phi_{ij}∼n\left(\frac{\sum_{i\;j}\phi_{ij}}{d_{i}},\frac{\sigma_{i}^{2}}{d_{i}}\right) \end{array}$$

where $\sigma_{i}^{2}$ is unknown spatial variance, and the joint probability density for $\phi_{i}$ is centered at 0 and has precision matrix *Q*. Assuming variance $\sigma^{2}=1$, the joint specification becomes;

(A5) $$\begin{array}{c} p\left(\phi \right)=exp(\frac{-1}{2}\sum_{i\;j}{\left(\phi_{i}-\phi_{j}\right)}^{2}) \end{array}$$

This is a pairwise difference formulation, each each $\left(\phi_{i}-\phi_{j}\right)$ is only dependent on the distance between the values of the neighboring regions. Adding $\sum_{N}\phi_{i}=0$ centers this model, and the log probability of this model is constrained to be between 0 and 1.

The precision matrix is constructed from the adjacency matrix and the diagonal matrix; the two matrices describe the neighborhood structure of a region *P*. The adjacency matrix is an PXP matrix where every entry $p_{ij}=1$ if regions $p_{i}$ and $p_{j}$ are neighbours and 0 otherwise. The diagonal matrix is a PXP matrix the diagonal elements $p_{ii}$ contain the number of neighbors of each region $p_{i}$ and all the off-diagonal entries are 0. The specification for the precision matrix is therefore;

(A6) Q=D(I−A)

The determinant of this matrix is 0, but with some data from *N* it is positive definite. The advantage of the ICAR model from the usual CAR model is the reduction of computation time, and capturing the spatial dependence mainly by the distance between the values of the neighboring regions.

### Appendix A.5. Bayesian Approach

For an estimation of the model parameters, a Bayesian approach is used and assigned priors for the model priors, and the distribution of the baseline survival function. An Intrinsic Conditional auto-regressive (ICAR) prior was assumed to model the spatial structure [3]. The prior models the mean for each area $\phi_{i}$, which is conditional on areas neighboring it; is normally distributed with mean equal to the average of the neighboring areas $\phi_{i}$ and variance is inversely proportional to the number of neighbors $d_{i}$ as above.

For the semi-parametric survival function, the baseline survival function is modeled using a Transformed Bernstein Polynomial (TBP) prior, usually centered around a given parametric family and selects only smooth densities [22]. The prior $TBP_{L}\left(\alpha ,S_{\theta }\left(\cdot \right)\right)$, where α>0 and $S_{\theta }$ are parameters of a Dirichlet Process, defined as

(A7) $$S_{0}\left(t\right)=\sum_{j=1}^{L}w_{j}I\left(S_{\theta }\left(t\right)|j,L-j+1\right),$$

where $W_{L}∼Dirichlet\left(\alpha ,\cdots ,\alpha \right)$, and $W_{L}={\left(w_{1}.\cdots ,w_{L}\right)}^{T}$ is a vector of positive weights, I(·|a,b) denotes a beta cumulative distribution function with (a,b) as parameters and $S_{\theta }\left(\cdot \right):\theta \in \Omega$ is a parametric family of survival functions. The log-logistic

(A8) $$S_{\theta }\left(t\right)={\left(1+{\left(e^{\theta_{1}}t\right)}^{exp(\theta_{2})}\right)}^{-1}$$

is chosen as the prior specification for the baseline survival function in this study. Vague normal priors are chosen for the regression coefficients, and a gamma prior $\tau^{-2}∼\Gamma \left(a_{\tau },b_{\tau }\right)$ for the precision parameter [23]. Posterior inference on the parameters were computed using Markov Chain Monte Carlo iterations carried out through a Bayesian approach [23]. The likelihood function based on $\left(w_{L},\theta ,\beta ,\phi \right)$ is given by;

(A9) $$L\left(w_{L},\theta ,\beta ,\phi \right)=\prod_{i=1}^{k}\prod_{j=1}^{n_{i}}{\left[Sx_{ij}\left(a_{ij}\right)-Sx_{ij}\left(b_{ij}\right)\right]}^{I(a_{ij}\leq b_{ij})}fx_{ij}{\left(a_{ij}\right)}^{I(a_{ij}=b_{ij})}$$

The posterior distribution given as

(A10) $$\begin{array}{c} p\left(w_{L},\theta ,\beta ,\phi \right)=p\left(\theta ,\beta ,\phi \right)L\left(w_{L},\theta ,\beta ,\phi \right) \end{array}$$

where p(θ,β,ϕ) is the prior distribution of the parameters and $L(w_{L},\theta ,\beta ,\phi )$ is the likelihood function obtained from the observed values.

Model comparison is done using the Deviance Information Criterion (DIC). Analysis was carried out in the R-software for statistics with the spBayesSurv package. A burn-in period of 5000 iterations was considered, and displayed after every 1000 saved iterates and run for 615 s on an HP Intel Core i3 Laptop computer. Small values of DIC indicated better performing models.

The flow diagram for the model implementation is shown in Figure A1.
